# Supplementary material for: Natural hybridization in heliconiine butterflies: the species boundary as a continuum
Source: BMC Evol Biol. 2007 Feb 23;7:28. doi: 10.1186/1471-2148-7-28 (PMC1821009; doi:10.1186/1471-2148-7-28)
Supplement: Additional File 1 — Hybrids between species of Heliconius and Eueides butterflies: a database. HTML file linking to database of all known wild-caught interspecific hybrid specimens in the Heliconiina, consisting of introductory text, a list of specimens, together with collection data and photographs of the specimens, and links to information about some artificial hybrids and mutants in the group. This is an edited copy of our online database of Heliconius hybrids [102]. To view database, download zip file and extract to a separate folder, then open index.html within that folder. [file 1471-2148-7-28-S1.zip › hcscly01.html]

hybrid hcscly01


---


Hybrid between *Heliconius clysonimus montanus* and *H. hecalesia formosus*
Costa Rica
© L. Gilbert

Return to table
of hybrids

To next hybrid (NONE!)
  
To previous hybrid

```
NOTES

No:                      161
Genus of species 1:      Heliconius
Species 1:               hecalesia
Subspecies of species 1: formosus
Genus of species 2:      Heliconius
Species 2:               clysonymus
Subspecies of species 2: montanus
Sex:                     m
Country:                 Costa Rica
Locality:                Cartago: Jicotea, Turrialba 1100m
Year:                    1995
Photo no.:               hcscly01
Named hybrid:
Collection:              MNCR
Collector:               G. Vega, A. Valerio
Author/publication:
Notes:                   F1
```

**Last updated:** 18 October 2003

---
